# Frontiers and hotspots evolution in mild cognitive impairment: a bibliometric analysis of from 2013 to 2023

**DOI:** 10.3389/fnins.2024.1352129

**Published:** 2024-08-16

**Authors:** Chunying He, Xiaohua Hu, Muren Wang, Xiaolan Yin, Min Zhan, Yutong Li, Linjuan Sun, Yida Du, Zhiyan Chen, Huan Wang, Haibin Shao

**Affiliations:** ^1^Department of Neurology, China Academy of Chinese Medical Sciences Xiyuan Hospital, Beijing, China; ^2^Graduate School, China Academy of Chinese Medical Sciences, Beijing, China; ^3^Department of Gastroenterology, China Academy of Chinese Medical Sciences Xiyuan Hospital, Beijing, China; ^4^Graduate School, Beijing University of Traditional Chinese Medicine, Beijing, China

**Keywords:** mild cognitive impairment, bibliometric analysis, visualized analysis, CiteSpace, VOSviewer

## Abstract

**Background:**

Mild cognitive impairment is a heterogeneous syndrome. The heterogeneity of the syndrome and the absence of consensus limited the advancement of MCI. The purpose of our research is to create a visual framework of the last decade, highlight the hotspots of current research, and forecast the most fruitful avenues for future MCI research.

**Methods:**

We collected all the MCI-related literature published between 1 January 2013, and 24 April 2023, on the “Web of Science.” The visual graph was created by the CiteSpace and VOSviewer. The current research hotspots and future research directions are summarized through the analysis of keywords and co-cited literature.

**Results:**

There are 6,075 articles were included in the final analysis. The number of publications shows an upward trend, especially after 2018. The United States and the University of California System are the most prolific countries and institutions, respectively. Petersen is the author who ranks first in terms of publication volume and influence. Journal of Alzheimer’s Disease was the most productive journal. “neuroimaging,” “fluid markers,” and “predictors” are the focus of current research, and “machine learning,” “electroencephalogram,” “deep learning,” and “blood biomarkers” are potential research directions in the future.

**Conclusion:**

The cognition of MCI has been continuously evolved and renewed by multiple countries’ joint efforts in the past decade. Hotspots for current research are on diagnostic biomarkers, such as fluid markers, neuroimaging, and so on. Future hotspots might be focused on the best prognostic and diagnostic models generated by machine learning and large-scale screening tools such as EEG and blood biomarkers.

## 1 Introduction

Mild cognitive impairment (MCI) is a clinical syndrome in which one or more cognitive aspects are impaired but daily activities are not significantly affected (Huszár et al., 2024). Regarded as a symptomatic prodromal phase of dementia, mild cognitive impairment (MCI) may progress to various forms of dementia over the next five years. One possible explanation for the recurring setbacks in the development of dementia medications could be irreversible brain damage throughout the dementia stage. Scholars are focusing more on MCI since it could be a significant target without irreversible neurological damage (Dunne et al., 2021). Over the past decade, researchers have made significant strides, particularly in the domains of MCI biomarkers, related illnesses, and risk factors. These valuable discoveries explored various underlying pathophysiological mechanisms, provided more objective diagnostic techniques, and presented a chance to lower risk and alter harmful behaviors (Ou et al., 2020; Yang et al., 2023).

Although some progress has been made in the past 10 years, several factors still hinder its further development. Despite a plethora of research about MCI interventions has been conducted such as diet, exercise, cognitive stimulation activities, and medication, there is still no approved treatment for MCI (Lissek et al., 2024). Besides, The connotation of MCI has evolved in the past 10 years mainly including subtypes and diagnostic criteria. Since the lack of unified diagnostic criteria, validated operationalized process flow, and standardized screening tools, the prevalence of MCI varied sharply from 10 to 74% in different studies (Jia et al., 2020; Tahami Monfared et al., 2022). “MCI” is a general term, various subtypes of MCI are very different in etiologies, underlying pathophysiological processes, and prognostic outcomes (Ashtari-Majlan et al., 2022; Gonuguntla et al., 2022). Numerous risk factors and related disorders of MCI have been found such as hypertension, stroke, diabetes, depression, sarcopenia, etc. However, there is a dispute about whether the apolipoprotein E allele is or not the risk factor for MCI between different researches (Hu et al., 2022; Cong et al., 2023). Consequently, the heterogeneity of the syndrome and the absence of consensus limited the advancement of MCI. As hundreds of documents emerge every year, it is quite challenging to assess the publication quality and determine which direction is the most prospective.

Bibliometric analysis is a quantitative tool for analyzing and integrating data to build a visible knowledge structure. It is widely employed for evaluating the quantity, quality, and correlation of publications (Wilson et al., 2021). Compared to traditional systematic review, bibliometric analysis offers the advantages of transparency and reproducibility, which enable researchers to map research areas objectively. In comparison to meta-analysis, it has the advantages of visualization and offers insight into the emergence, evolution, and potential future study directions of a field. These benefits have led to the widespread use of bibliometrics in several study fields for structure mapping and literature reviews (Gan et al., 2022). The purpose of our research is to create a visual framework that maps the trajectory of MCI during the last ten years, highlights the hotspots of current research, and forecasts the most fruitful avenues for future MCI research to identify a critical breakthrough.

## 2 Materials and methods

### 2.1 Data collection strategy

**FIGURE 1:**
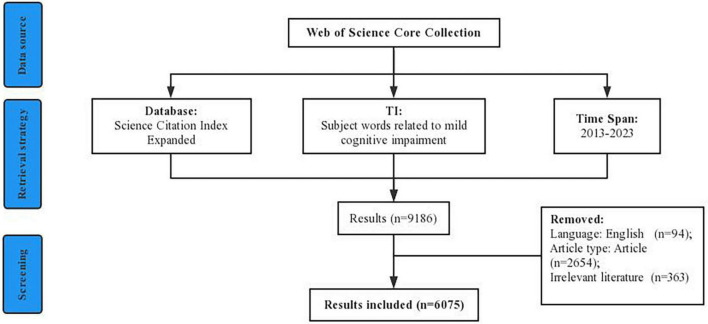
Flowchart of literature screening.

The Web of Science (WoS), an authoritative database that could provide the overall data source, was widely used for search data. In this study, data were obtained from the Science Citation Index Expanded (SCI-E) database within the “Web of Science Core Collection” (WoSCC) between 1 January 2013 and 24 April 2023. The search strategy was: TI = (“MCI*” OR “Mild Cognitive Impairment*” OR “Mild Neurocognitive Disorder*” OR “Mild cognitive*” OR “Mild Cognitive dysfunction*”) AND Language = (“English”) AND Document Type = (“Article”). After manually reading the titles and abstracts, irrelevant documents were excluded, and a total of 6,075 documents finally met the inclusion criteria and were included. Then the data was imported into the CiteSpace software for checking duplicates. To get comprehensive information, we use the mode “Full Record and Cited References.” These retrieved records were saved as “plain text” file formats and named download_txt. All data collection was conducted on 24 April 2023. In addition, Citations, Impact factor (IF), and H-index were used to supplement. Figure 1 depicts the flow chart of the literature screening.

### 2.2 Data analysis

The full data was analyzed using CiteSpace 6.2.4 and VOSviewer software 1.6.19. VOSviewer 1.6.19 was used for institutions, authors, and co-cited authors, journals, and Keyword analyses. CiteSpace 6.2.4 was used for presentation visualization analysis of journals, co-cited references, and keywords. SCImago Graphica 1.0.36 was used for performing the cooperative relations between countries/regions. Bibliometrix R Package software 4.3.0 was used to display source dynamics and topic dynamics.

The basic parameters of CiteSpace were set as follows: (1) time slicing (2013–2023), year per slice for 1 year; (2) for the text processing section, set the term source to Title, Abstract, Author, and Keywords; (3) node type was selected at a time. The parameters of VOSviewer were set as below: (1) set the analysis type to co-occurrence; (2) the counting method was set as full counting; (3) according to data analysis requirements, select different visualization maps such as network, overlay, and density visualization.

## 3 Results

### 3.1 Annual publication outputs

**FIGURE 2:**
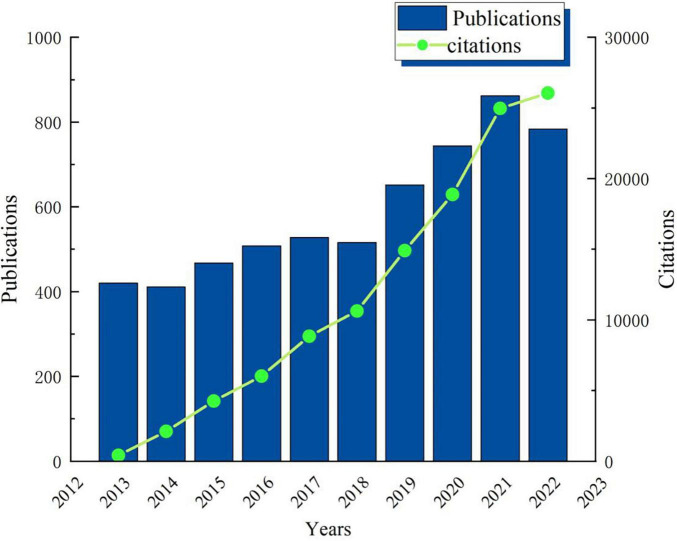
Annual global growth trends in the field of mild cognitive impairment from 2013 to 2022.

According to the Citation Report of WoS, the total citation number of the 6,075 documents was 123,493, with an average of 20.33 citations per article, and the H-index of was 116. During the past decade, dynamic change in the annual global publications shows a two-stage trend (Figure 2). Stage 1: during the period from 2013 to 2018, the annual publication volume fluctuated steadily between 410 and 530, and the number of publications each year was relatively stable. Stage 2: during the period from 2018 to 2022, the number of papers published each year has greatly increased, with an average annual increase of about 100 articles. The overall upward trend of citation number, increasing frequency of publications, and high H-index show that scholars pay more and more attention to the field of mild cognitive impairment.

### 3.2 Contribution of countries/regions

**FIGURE 3:**
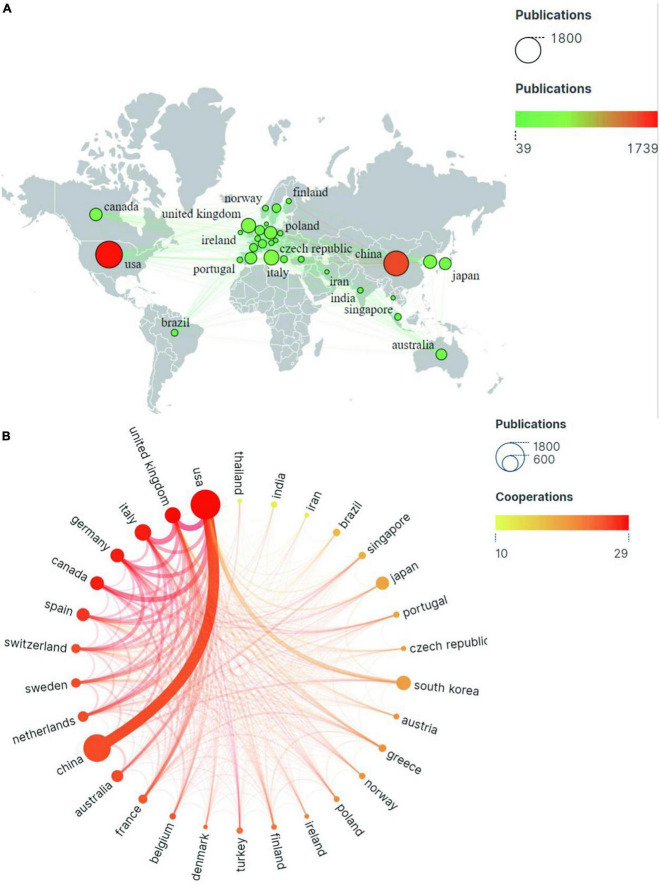
The publications and partnership strength of countries/regions in the field of mild cognitive impairment. **(A)** Geographical heat map of publications. **(B)** Partnership chord chart for the 30 most productive countries/regions.

There are 95 countries involved in the study of mild cognitive impairment. As the two most prolific countries, the United States in North America topped the list with 1,739 publications, followed by China in Asia with 1,479 publications. These two countries alone account for half of all global publications. Otherwise, productive countries are mostly concentrated in Europe (Figure 3A).

The circles in Figure 3B represent countries/regions, and their size correlates with the volume of publications. The color of the line is related to the strength of cooperation, with red representing the country/region’s important position in the network of international cooperation. As is shown in the chart, the United States ranks first in terms of production and cooperation intensity. Although Italy (538 articles) and Germany (372 articles) do not rank very high in terms of output, they rank second and third, respectively, in terms of international partnerships. China ranked second in the number of articles published, but 10th in international cooperation (Table 1). This may be related to the geographical location and the complex flow of research.

**TABLE 1:** The top 10 productive countries/regions and institutions in the field of mild cognitive impairment ranked by publication number.

Ranking	Country	Count (percentage)	Centrality	Average citations	H-index
1	USA	1739 (27.32%)	0.17	27.24	94
2	China	1479 (23.24%)	0.05	14.31	57
3	Italy	538 (8.45%)	0.15	22.20	55
4	The United Kingdom	495 (7.78%)	0.22	27.71	56
5	South Korea	415 (6.52%)	0.03	18.79	43
6	Germany	372 (5.84%)	0.17	27.96	52
7	Canada	372 (5.84%)	0.12	26.59	50
8	Japan	346 (5.44%)	0.1	18.84	39
9	Spain	330 (5.18%)	0.1	20.57	42
10	Australia	279 (4.38%)	0.12	25.70	44

USA, the United States of America.

The H-index is one of the indicators to evaluate influence. The United States ranks first with the 94 H-index. There is little difference in the H-index among China, the United Kingdom, and Italy, ranking second, third, and fourth, respectively. Germany, the United Kingdom, and the United States rank among the top three in terms of average citations per paper, while China ranked 10th with 14.31. This shows that the Occident has a higher influence.

### 3.3 Contribution of institutions

**FIGURE 4:**
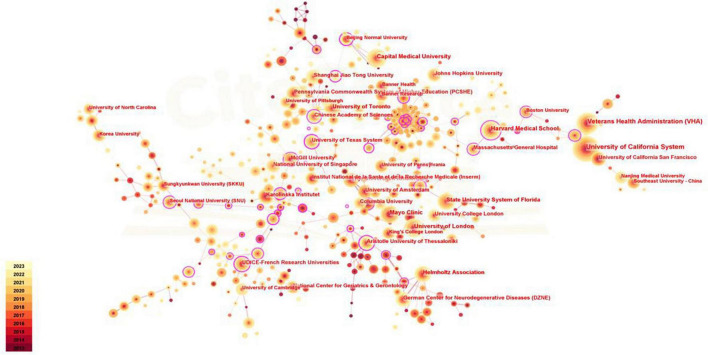
The collaboration of institutions in the field of mild cognitive impairment.

A total of 6,198 institutions worldwide have contributed to this field over the past 10 years, of which the University of California System ranks first in the high-yield list with 277 publications. Among the top ten most productive institutions, six belong to the United States, which shows the amazing academic dominance of the United States in this field. The institution’s average citations per article of 56 far exceeds the second place, but it ranks second on the H-index list, which may be due to the small number of articles published (Table 2). The purple outer circle of the node in Figure 4 represents high centrality, as shown in the figure, most of the highly centralized countries are located in Europe and America, especially in the United States, such as Boston University, Albany Medical College, and Harvard Medical School. Additionally, there is tight collaboration and scholarly interchange among European and American nations, particularly in highly productive countries, such as the University of California System, Veterans Health Administration, and Mayo Clinic. In addition, there are some highly cooperative institutions located in China, but more show domestic cooperation, such as Capital Medical University, Beijing Normal University, Chinese University of Hong Kong, and Chinese Academy of Sciences.

**TABLE 2:** The top 10 institutions that contributed publications in the field of mild cognitive impairment from 2013 to 2023.

Ranking	Institutions (country)	Count (percentage)	Centrality	Average citations	H-index
1	University of California System (USA)	277 (17.79%)	0.05	31.74	50
2	Veterans Health Administration (USA)	209 (13.42%)	0.03	25.92	41
3	University of London (UK)	156 (10.02%)	0.02	30.63	39
4	Capital Medical University (CHINA)	156 (10.02%)	0.07	18.33	29
5	Harvard Medical School (USA)	154 (9.89%)	0.03	27.23	36
6	Mayo Clinic (USA)	135 (8.67%)	0.01	56.61	41
7	University of Toronto (CANADA)	128 (8.22%)	0.05	23.77	30
8	State University System of Florida (USA)	123 (7.9%)	0.04	24.67	27
9	Helmholtz Association (GERMANY)	118 (7.58%)	0.02	28.11	33
10	Johns Hopkins University (USA)	101 (6.49%)	0.07	33.53	34

USA, the United States of America; UK, the United Kingdom.

### 3.4 Authors and co-cited authors

**FIGURE 5:**
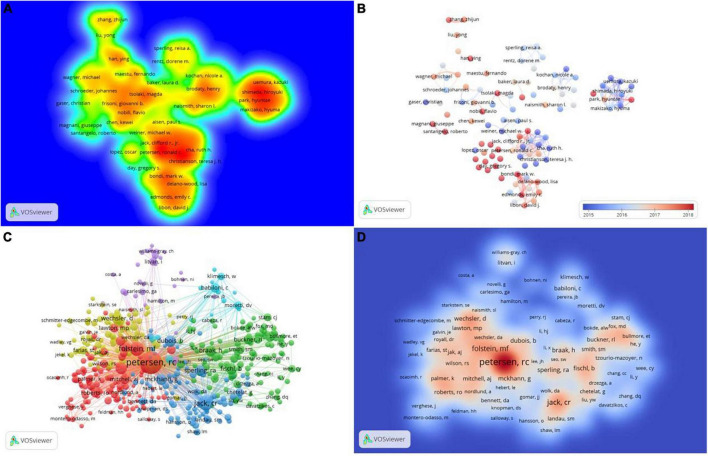
The collaboration of authors and co-cited authors in the field of mild cognitive impairment. **(A)** Heat map of the author’s published volume. **(B)** The time-overlay map of the cooperation network among the authors. **(C)** Cooperation network among the co-cited authors. **(D)** Heat map of the co-cited author’s published volume.

In the past 10 years, a total of 25,944 authors have been devoted to the study of mild cognitive impairment. In this study, authors cited more than 500 times were defined as core researchers and a total of 105 authors were finally considered as core researchers. As shown in Table 3, only two of the top 10 prolific authors are from Asia, while the rest are from Europe and the United States. Figure 5A clearly shows the difference between the authors’ volume of postings. Combined with the documents and the impact index, Petersen, Ronald C, Shen, Dinggang, and Knopman, David S played a key role in this field. Figure 5B shows a superimposed annual view of the core researcher collaboration network. Collaborations between authors are mostly concentrated from 2015 to 2018, with red representing newer collaborations.

**TABLE 3:** Top 10 productive authors and co-cited authors in the field of mild cognitive impairment from 2013 to 2023.

Ranking	Author	Institution (country)	Count (percentage)	Average citations	H-index	Co-cited author	Institution (country)	Co-citations	H-index
1	Petersen, Ronald C	(Mayo Clinic, USA)	55 (12.25%)	94.2	32	Petersen, Ronald C	(Mayo Clinic, USA)	6,267	116
2	Tsolaki, Magda	(Aristotle Univ Thessaloniki, Greece)	53 (11.80%)	35.77	25	Jack, Cr	(Mayo Clinic, USA)	1,816	119
3	Frisoni, Giovanni B	(IRCCS Ctr S Giovanni di Dio FBF, Italy)	45 (10.02%)	37.82	22	Frisoni, Giovanni B	(IRCCS Ctr S Giovanni di Dio FBF, Italy)	1,719	61
4	Shen, Dinggang	(Univ North Carolina Chapel Hill, USA)	44 (9.80%)	66.39	26	Albert, Ms	(Johns Hopkins University, USA)	1,592	38
5	Knopman, David S	(Mayo Clinic, USA)	44 (9.80%)	72.16	28	Morris, Jc	(Washington University School of Medicine, USA)	1,454	101
6	Shimada, Hiroyuki	(Natl Ctr Geriatr & Gerontol, Japan)	44 (9.80%)	37.16	24	Winblad, B	(Karolinska Institutet, Sweden)	960	63
7	Bondi, Mark W	(Vet Affairs San Diego Healthcare Syst, USA)	42 (9.35%)	41.12	21	Dubois, B	(Assistance Publique Hopitaux Paris, France)	886	61
8	Mielke, Michelle M	(Mayo Clinic, USA)	41 (9.13%)	61.66	19	Mckhann, G	(Johns Hopkins School of Medicine, USA)	828	6
9	Nobili, Flavio	(Univ Genoa, Italy)	41 (9.13%)	30.29	18	Nasreddine, Zs	(MoCA Clinic and Institute, Canada)	778	8
10	Han, Ying	(Beijing Inst Brain Disorders, China)	40 (8.91%)	21.68	14	Braak, H	(Ulm University, Germany)	698	15

USA, the United States of America.

The top 10 authors with the most co-cited times are all from European and American countries, among which 5 are from the United States and 2 are from the same institution: Mayo Clinic. Petersen, Ronald C far exceeds other authors with a total of 6,267 co-citations, which shows his outstanding position in this field. According to their co-citation relationship, co-cited authors are divided into six categories (Figure 5C). Figure 5D shows the influence of co-cited authors in this field.

### 3.5 Journal and co-cited journals

**FIGURE 6:**
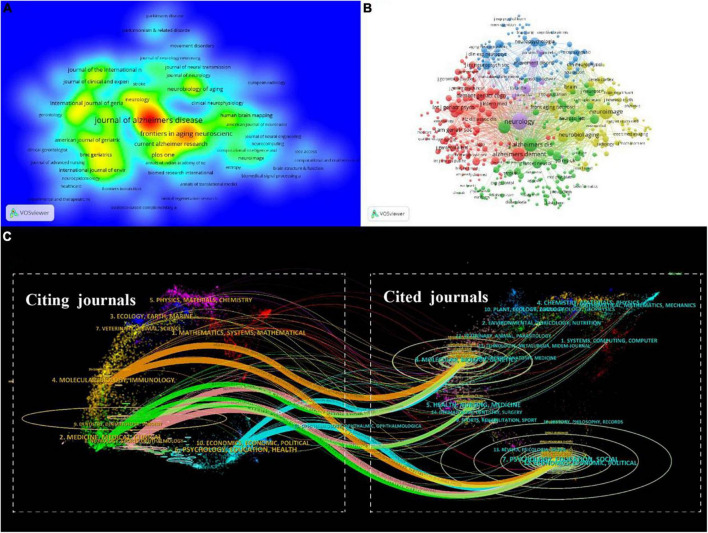
The Journal analyses in the field of mild cognitive impairment. **(A)** Density map of citing journals based on the number of publications. **(B)** VOSviewer network visualization map of the co-citation of journals. **(C)** The dual-map overlay of journals in the field of mild cognitive impairment.

A total of 806 journals reported studies on mild cognitive impairment, and 15,979 journals were cited by these journals. As shown in Figure 6A, the Journal of Alzheimer’s Disease was the most productive journal (714, 37.58%), followed by Frontiers in Aging Neuroscience (313, 16.47%) and PLoS One (129, 6.79%). The top ten journals are listed in Table 4, most of them are from Europe and America, of which three belong to the United States and three belong to the United Kingdom. Four journals have an impact factor > 5, including Frontiers in Aging Neuroscience (5.702), Alzheimer’s & Dementia (16.655), International Psychogeriatrics (7.191), and Neurobiology of Aging (5.133).

**TABLE 4:** Top 10 prolific journal in mild cognitive impairment.

Ranking	Journal	Country (publisher)	Publications (%)	Average citations	Impact factor (2022)	JCR	SCImago journal rank (2022)	H-index
1	Journal of Alzheimer’s Disease	(Netherlands, IOS PRESS)	714 (37.58%)	19.17	4.16	Q2	1.15	52
2	Frontiers in Aging Neuroscience	(Switzerland, Frontiers Media S.A.)	313 (16.47%)	14.47	5.702	Q1	1.21	36
3	PLoS One	(United States, Public Library of Science)	129 (6.79%)	30.74	3.752	Q2	0.89	35
4	Current Alzheimer Research	(United Arab Emirates, Bentham Science Publishers B.V.)	117 (6.16%)	18.87	3.04	Q3	0.64	27
5	International Journal of Geriatric Psychiatry	(United Kingdom, John Wiley and Sons Ltd.)	100 (5.26%)	18.37	3.85	Q2	1.23	25
6	Alzheimer’s & Dementia	(United States, John Wiley & Sons Inc.)	99 (5.21%)	41.07	16.655	Q1	3.29	37
7	Dementia and Geriatric Cognitive Disorders	(Switzerland, S. Karger AG)	97 (5.11%)	16.48	3.346	Q3	0.75	25
8	International Psychogeriatrics	(United Kingdom, Cambridge University Press)	95 (5.00%)	16.67	7.191	Q1	1.25	21
9	Neurobiology of Aging	(United States, Elsevier Inc.)	86 (4.53%)	28.01	5.133	Q2	1.52	29
10	Scientific Reports	(United Kingdom, Nature Publishing Group)	76 (4.00%)	20.20	4.997	Q2	0.97	23

The foundation of current research frontiers is earlier research that was published in co-cited publications. The co-citation analysis was conducted by the VOSviewer (Figure 6B). In the top ten co-cited journals, five are from the United States and three are from the United Kingdom, which shows the high academic influence of these two countries. Besides six have an impact factor > 5 and five belong to Q1, demonstrating the high quality of co-cited journals (Table 5).

**TABLE 5:** Top 10 co-cited journals in mild cognitive impairment.

Ranking	Co-cited journal	Country (publisher)	Co-citations	Impact factor (2022)	JCR	SCImago journal rank (2022)	H-index
1	Neurology	(United States, Lippincott Williams and Wilkins Ltd.)	14,333	11.8	Q1	2.54	396
2	Neuroimage	(United States, Academic Press Inc.)	10,205	7.4	Q1	2.51	399
3	Alzheimer’s & Dementia	(United States, John Wiley & Sons Inc.)	8,715	16.655	Q1	3.29	150
4	Journal of Alzheimer’s Disease	(Netherlands, IOS PRESS)	8,656	4.16	Q2	1.15	163
5	Neurobiology of Aging	(United States, Elsevier Inc.)	5,860	5.133	Q2	1.52	205
6	Brain	(United Kingdom, Oxford University Press)	4,451	15.255	Q1	4.44	365
7	PLoS One	(United States, Public Library of Science)	4,160	3.752	Q2	0.89	404
8	Dementia and Geriatric Cognitive Disorders	(Switzerland, S. Karger AG)	3,879	3.346	Q3	0.75	120
9	Journal of the American Geriatrics Society	(United Kingdom, Wiley-Blackwell Publishing Ltd.)	3,701	7.538	Q1	2.05	254
10	International Journal of Geriatric Psychiatry	(United Kingdom, John Wiley and Sons Ltd.)	3,417	3.85	Q2	1.23	143

**FIGURE 7:**
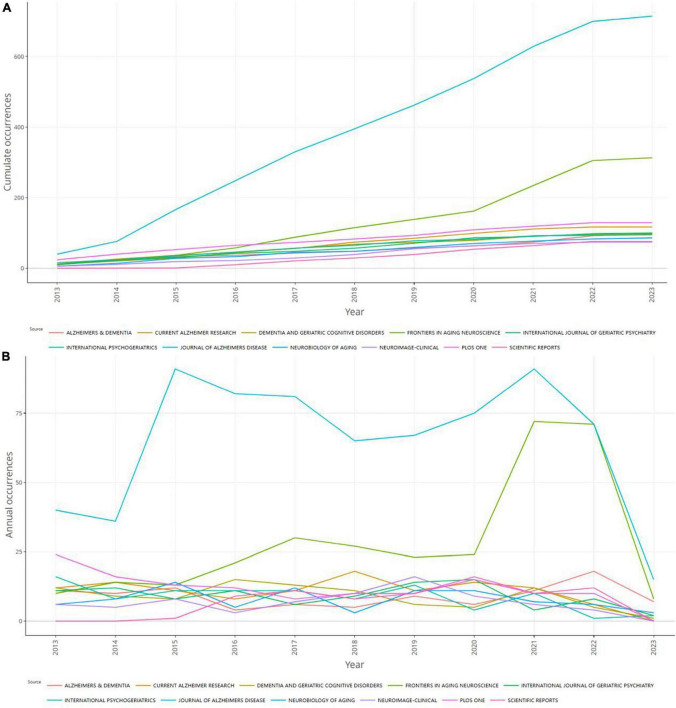
Source dynamics in the field of mild cognitive impairment. **(A)** Cumulate occurrences. **(B)** Annual occurrences.

The dual-map overlay of journals refers to the overlaying of citing and cited journals on a single graph to investigate the relationship between journals. The left side of the graph is the citing journal, the right side is the cited journal, and the color path represents the citation relationship in Figure 6C. The research of citing journals mainly refers to (1) molecular, biology, and immunology; (2) medicine, medical, and clinical; (3) neurology, sports, ophthalmology; (4) psychology, education, health, while most publications of cited journals come from (1) molecular, biology, genetics; (2) health, nursing, medicine; (3) psychology, education, health. This shows that genetics, fundamental medical fields, psychological fields, and clinical fields are all tightly related to MCI. The source dynamics are presented in Figure 7 displaying the cumulate and annual occurrences of the 10 journals with the largest number of publications in this field.

### 3.6 Co-cited references and references burst

Co-cited reference refers to being cited by other publications at the same time. These documents are inextricably linked to form a co-cited literature network, which is an invaluable tool for analyzing high-quality research dynamics in this field (Effah et al., 2023). Co-citation reference analysis helps grasp the research trends and high-quality research progress in this field and related research fields. A total of 122,253 articles were included in the analysis, and the top 10 most-cited articles are listed in Table 6. The guideline “The diagnosis of mild cognitive impairment due to Alzheimer’s disease: recommendations from the National Institute on Aging-Alzheimer’s Association workgroups on diagnostic guidelines for Alzheimer’s disease” in the Alzheimer’s & Dementia (IF: 16.655) ranked first with 474 co-citations. Among the highly co-cited references, the most popular topics included the diagnosis, biomarkers, definition, and clinical guidelines of Alzheimer’s disease and mild cognitive impairment. Almost Half of the top ten co-cited documents were published in Alzheimer’s Dementia (IF: 16.655), which is the most prestigious top journal in this field. In addition, Lancet (IF: 202.731) and Lancet Neurology (IF: 59.935) are also very authoritative among peer-reviewed journals.

**TABLE 6:** Top 10 highly cited publications with mild cognitive impairment.

Ranking	Title	Citation	Type	Journal	Country/region	Year	Impact factor (2022)
1	The diagnosis of mild cognitive impairment due to Alzheimer’s disease: recommendations from the National Institute on Aging-Alzheimer’s Association workgroups on diagnostic guidelines for Alzheimer’s disease	474	Guideline	Alzheimer’s Dementia	USA	2011	16.655
2	NIA-AA Research Framework: toward a biological definition of Alzheimer’s disease	289	Review	Alzheimer’s Dementia	USA	2018	16.655
3	Practice guideline update summary: mild cognitive impairment: report of the Guideline Development, Dissemination, and Implementation Subcommittee of the American Academy of Neurology	258	Guideline	Neurology	USA	2018	11.8
4	The diagnosis of dementia due to Alzheimer’s disease: recommendations from the National Institute on Aging-Alzheimer’s Association workgroups on diagnostic guidelines for Alzheimer’s disease	193	Guideline	Alzheimer’s Dementia	USA	2011	16.655
5	Mild cognitive impairment: a concept in evolution	153	Review	J Intern Med	USA	2014	13.068
6	Toward defining the preclinical stages of Alzheimer’s disease: recommendations from the National Institute on Aging-Alzheimer’s Association workgroups on diagnostic guidelines for Alzheimer’s disease	152	Guideline	Alzheimer’s Dementia	USA	2011	16.655
7	Dementia prevention, intervention, and care	133	Review	Lancet	UK	2017	202.731
8	Advancing research diagnostic criteria for Alzheimer’s disease: the IWG-2 criteria	119	Review	Lancet Neurology	France	2014	59.935
9	Diagnostic criteria for mild cognitive impairment in Parkinson’s disease: Movement Disorder Society Task Force guidelines	109	Guideline	Movement Disorder	USA	2012	9.698
10	Diagnostic and statistical manual of mental disorders	104	Review	Psychiatry Research	USA	2011	11.225

USA, the United States of America; UK, United Kingdom.

**FIGURE 8:**
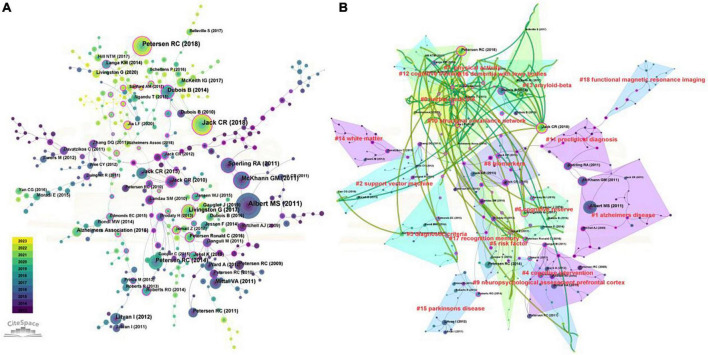
Co-cited reference analyses in the field of mild cognitive impairment. **(A)** The time-overlay map of the co-cited references networks. **(B)** The dual-map overlay of co-cited reference in the field of mild cognitive impairment.

As the illustration in Figure 8A shows, different colors represent the corresponding year, and the connection represents the co-citation relationship. After cluster analysis, this literature was divided into 18 categories, such as Alzheimer’s disease (#1), diagnostic criteria (#3), biomarkers (#8), and functional nuclear magnetic resonance (#18). In order to better predict the future research direction and understand the current research topics of mild cognitive impairment, the cluster map of co-cited documents from 2022 to 2023 was superimposed on the original map (Figure 8B). The categories covered by the green line represent areas where the latest research is still being devoted, such as Herbal medicine (#0), physical activity (#7), cognitive training (#12), and amyloid-beta (#13).

### 3.7 Keyword analysis

**FIGURE 9:**
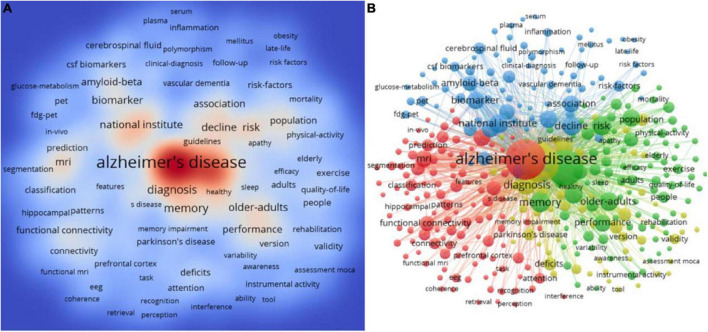
Keyword analyses in the field of mild cognitive impairment. **(A)** Density visualization of keywords. **(B)** Cluster visualization of keywords.

Keyword analysis can reflect the evolution and hotspots of a research field, especially the frontiers of research. According to the high-frequency keywords of the co-occurrence network, the four keywords “Alzheimer’s disease,” “mild cognitive impairment,” “dementia,” and “diagnosis” ranked the top four with a total occurrence of 4,040 times, 3,497 times, 3,121 times and 736 times, respectively (Figure 9A), which revealed that mild cognitive impairment was closely related to dementia and Alzheimer’s disease, and highlighted the importance of early diagnosis of mild cognitive impairment. In addition, the high-frequency keywords of the co-occurrence network are divided into 4 categories through cluster analysis (Figure 9B). #1: Neuroimaging is an important diagnostic tool for mild cognitive impairment, which currently includes magnetic resonance imaging (MRI), PET, single-photon emission computed tomography (SPECT) imaging, and so on (Red cluster including “hippocampus,” “functional magnetic resonance,” “cortical thickness,” “structural MRI,” “white matter hyperintensities,” and “electroencephalography”); #2: Biomarkers have become an indispensable diagnostic criterion for mild cognitive impairment, and the two most widely studied markers are reflected Aβ deposition and tau deposition (Blue cluster including “amyloid-beta,” “biomarker,” “cerebrospinal fluid,” “clinical-diagnosis,” “plasma,” “progression,” and “tau”); #3: Cognitive tests are the important evidence for assessing the presence of objective cognitive decline, which plays a key role in the diagnosis of mild cognitive impairment (Yellow cluster including “accuracy,” “assessment moca,” “brief screening tool,” “cognitive screening,” “guidelines,” “mini-mental state examination,” and “neuropsychological tests”); #4: There are currently no approved pharmacologic for treating patients with MCI, but cognitive training and regular exercise has proved to be beneficial measures (Green cluster including “activities of daily living,” “aerobic exercise,” “caregivers,” “cognitive training,” “cognitive reserve,” “donepezil,” “efficacy,” and “intervention”).

**FIGURE 10:**
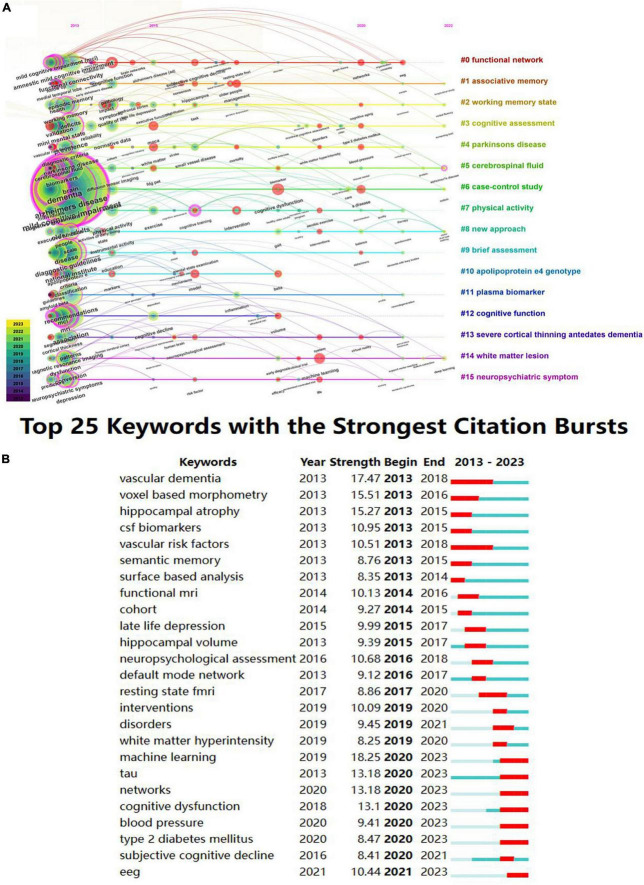
Keyword analyses in the field of mild cognitive impairment. **(A)** Timeline view of keyword clusters. **(B)** Top 25 keywords with the strongest citation bursts.

Burst keyword refers to a sudden spike in the frequency of keywords over a short period. By examining the burst keywords, research hotspots and frontiers in this topic can be identified (You et al., 2021). The red nodes in Figure 10A represent bursts of keyword citations. These hot topics included neuroimaging (functional magnetic resonance imaging, # 0), cognitive test (moca, # 3), classification (vascular dementia, # 4), biomarkers (tau, # 5), and treatment (intervention, # 7) of MCI. In the top 25 keywords with the strongest citation bursts over the past decades, machine learning ranked first with the highest burst strength (18.25), followed by vascular dementia (17.47), voxel based morphometry (15.51), hippocampal atrophy (15.27), and tau (13.18) (Figure 10B). The research of tau begins from 2013 and burst from 2020 to 2023. As is shown in the figure, machine learning (18.25), tau (13.18), networks (13.18), EEG (10.44), type 2 diabetes mellitus (8.47), is the current research hotspot.

## 4 Discussion

**FIGURE 11:**
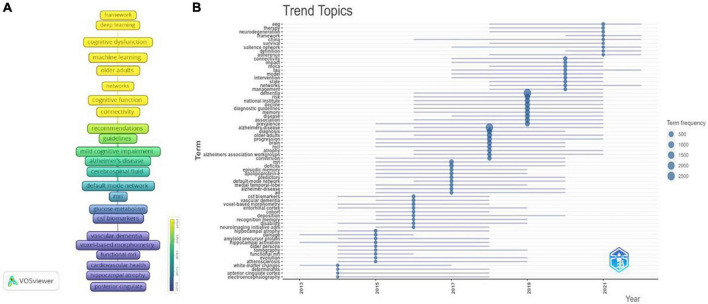
Keyword analyses in the field of mild cognitive impairment. **(A)** Timeline evolution diagram of keywords. **(B)** Topics dynamics in the field of mild cognitive impairment.

To show the evolution of keywords over time, the timeline of keywords is visualized in Figure 11A. Figure 11B shows the dynamic trend of topics. During the period of 2014–2015, the terms “anterior cingulate cortex,” “white matter changes,” and “hippocampal atrophy” revealed the most popular research on neuroimaging. During the period of 2016–2017, the terms “CSF biomarkers,” “deposition,” “predictors,” “apolipoprotein E,” “medial temporal lobe,” and “entorhinal cortex” suggested that hot theme was predictors of mild cognitive impairment progression to dementia, to more accurately identify high-risk groups; During the period of 2018–2019, the terms “prevalence,” “Alzheimer’s association work,” “older adults,” “diagnostic guidelines” suggested that the hottest topics at this stage were the diagnostic criteria of MCI and epidemiological studies. During the period of 2020–2023, the terms “management,” “intervention,” “moca,” “framework,” and “therapy” indicated that the attractive topic was the comprehensive clinical management of MCI, including neuropsychological assessment, intervention, diagnosis and treatment framework.

### 4.1 Overview of major findings

The number of annual publications in the first stage (2013–2018) showed a slow growth trend, the main reason may be that the concept of MCI is evolving. MCI started as a research term but gradually came into use in clinical practice. Due to the evolving concept of MCI and the lack of a unified consensus, there were no breakthroughs in the study of MCI at this stage (Petersen et al., 2014). Saunders et al. (2018) showed the direction of MCI research in 2018, and then the second phase grew rapidly. They thought that further research was needed in multi-domain biomarkers, more accurate neuroimaging techniques, more sensitive neuropsychological testing, and risk factors. Since then many studies have been conducted intensively based on these directions, and research on MCI has entered a phase of rapid development. The practice guidelines were updated in 2018 to guide and standardize clinical diagnosis and treatment of MCI and to facilitate advances in clinical practice (Petersen et al., 2018). A novel neuropsychological criterion has been proved that could improve the accuracy of MCI diagnosis in 2019, which further promoted the development of this field (Edmonds et al., 2019). Jia et al. (2020) proposed prevention strategies and integrated management in 2020 to reduce the prevalence of MCI and carry out early intervention, which was a beneficial exploration (Jia et al., 2020).

According to the collaborative network of countries/regions, institutions, and authors, the trend that the United States was far ahead, China was catching up, and Europe was an important center of scientific collaboration could be determined. The reason for the amazing dominance of the United States in the field of MCI is that many prominent authors as well as advanced research institutions are located in the US. The US is the maker of important concepts and diagnostic standards in the MCI field (Rabinovici et al., 2019). The deeper reason may be the highly developed economy of the United States to support research, the comprehensive education system to cultivate high-quality talents, and the favorable talent system to attract the gathering of talents. China’s high volume of publications and impact index may be attributed to its rapidly developing economy and the emphasis on MCI after entering an aging society. However, there is little international cooperation in China, which may be due to geographical constraints and a lack of creative research to break through academic barriers. A significant contribution to the development of the MCI field was made by the work of Petersen, and Ronald’s team on early biomarkers of MCI and risk factors for the progression of MCI to dementia. For example, Potential predictors like neuropsychiatric symptoms, olfactory deficits, and multiple metabolic pathways were identified (Geda et al., 2014; Roberts et al., 2016).

Alzheimer’s Dementia is regarded as one of the most significant journals based on the impact factor, the number of citations, and the H-index. The magazine, a leader in the field of cognitive science, is committed to publishing ground-breaking and exciting articles on a variety of subjects, including epidemiology, diagnostic criteria, and more (Bocchetta et al., 2015; Rajan et al., 2021; Festari et al., 2023).

### 4.2 Research hotspots and trends

Based on the literature co-citation and keyword analysis, the following promising novel studies in the field of MCI are proposed to guide valuable future research directions.

#### 4.2.1 Machine learning approach

Machine learning (ML) is a set of algorithms that can automatically, objectively, and efficiently detect complex data patterns in high-dimensional space, mainly including feature extraction and classification algorithms (Schäfer et al., 2023). There are numerous roadblocks that prevent the advancement of MCI research in this area, but ML may hold the key to overcoming these issues. (1) A heterogeneous disease like mild cognitive impairment is frequently accompanied by hazy, ambiguous information, but fuzzy logic algorithms in ML technology can handle this challenging situation (Rossini et al., 2022). (2) Numerous resources are available to assist in the diagnosis of MCI, including MRI, PET, blood and cerebrospinal fluid biomarkers, etc. It is impossible to recognize and manage it manually due to the enormous amount of data and intricate permutations. Relying on the enormous amount of computation, ML technology can process a vast quantity of complicated information rapidly, reduce the load on labor by humans, and be more objective and effective (Shah et al., 2023). (3) It is feasible to identify significant features that people haven’t noticed because ML can detect minor changes with greater objectivity and sensitivity. As a result, ML has the potential to find novel MCI biomarkers by overcoming the constraints of current human expertise (Ahmed et al., 2019). The advancement of MCI has been aided by the application of machine learning (ML) technology for dynamic changes in the pathophysiology, (Chang et al., 2021) diagnosis, (Liu et al., 2018) categorization, (Basaia et al., 2019; Jitsuishi and Yamaguchi, 2022) individualized risk assessment of conversion to AD, (Hojjati et al., 2018; Park et al., 2023) targeted therapy, and drug response assessment (Zhang et al., 2020). In conclusion, the rapid development of ML technology has opened up numerous opportunities and allowed for the resolution of numerous MCI-related issues.

#### 4.2.2 Multi-modal markers integration

The integration of multi-modal markers will become a popular research trend as a result of the immense complexity of brain function and structure making it impossible for a single modality to accurately display the brain information of MCI patients (Hinrichs et al., 2011). Through machine learning (ML), multimodal markers can be used to create a variety of models that incorporate many types of biomarkers, including information from population epidemiology, neuropsychology, and neuroimaging (Sapkota et al., 2018). Multimodal markers can improve data performance and provide complementary information, but in practice, features from different modalities are equally connected that can not explain their diversity (Chand et al., 2022; Karaman et al., 2022; Long et al., 2023). Therefore, specialized feature fusion strategies are urgently needed before ML training (Jiao et al., 2022).

#### 4.2.3 Electroencephalogram

Electroencephalogram (EEG) records the rhythmic discharge activity of brain neurons through the scalp and is recognized as being able to assess early functional connectivity disorders and synaptic pathology (Zhang et al., 2021). Synaptic transmission and functional connections are considered to be the initial targets of attack in the continuous progression of dementia (Ieracitano et al., 2020). Therefore, EEG can well reflect the early lesions in the continuous progression of AD and is used in MCI. For the early diagnosis and treatment of more MCI patients, research strategies should shift to a timely and public health-oriented approach (Spasov et al., 2019). Extend patient inclusion from memory clinic screening to the general public. To meet this strategic shift, four prerequisites need to be met: widely available, low-cost, non-invasive, and highly sensitive (Kim et al., 2023). The combination of EEG and ML technology fully meets these conditions and has great potential for future research.

#### 4.2.4 Deep learning

Deep learning is a branch of machine learning technology. It can create a multi-layer neural model network with a hierarchical and deep structure that is reminiscent of the human brain (Qiu et al., 2022). Deep learning algorithms are more effective than conventional ML techniques at handling high-dimensional, unstructured, and multi-modal data and extracting hidden features from the data (Jo et al., 2019). The major issue right now is that deep learning requires a lot of samples for training and fine-tuning parameters, yet the available samples are frequently insufficient (Zhou et al., 2021). Some research have used transfer learning to overcome the limits of using big volumes of medical data, and this problem can be solved by developing large-sample public databases, but this is still an area that requires special attention in the current application of deep learning technology (Fathi et al., 2022; Ghaffari et al., 2022).

#### 4.2.5 Functional near-infrared spectroscopy

Functional near-infrared spectroscopy (fNIRS) is another promising emerging technology that can be used for early MCI screening. This neuroimaging method employs infrared light rays emitted to the skull, with the dispersed rays collected by a photodetector that can measure the degree of light attenuation and absorption (Hayashi et al., 2024). fNIRS can detect differences in light wavelengths from oxy-hemoglobin (oxy-Hb) and deoxy-hemoglobin (deoxy-Hb), allowing it to identify neuron activity. In the course of cognitive decline, the earlier stage is typically characterized by hyper-perfusion (neurovascular compensatory mechanisms that require a greater metabolic level), whereas the later stage is characterized by hypo-perfusion (more severe neurodegeneration requires a lower metabolic level) (Liampas et al., 2024). Because fNIRS can quantify hemodynamic responses, many experts believe it has the potential to be an attractive method for early MCI diagnosis as well as long-term cerebral activity monitoring. fNIRS is a promising method for early screening and management of MCI due to its non-invasive, portable, low-cost, participant-friendliness, real-time feedback, and interoperability with other therapeutic devices (Bonilauri et al., 2020). fNIRS also has certain drawbacks, such as being limited to extracting absolute hemoglobin concentrations, measuring erratically with extracranial matter, and having lower spatial resolution that only reaches the outer cortex. Nonetheless, it is a promising novel neuroimaging method, and further technological developments are expected to fully realize its promise.

#### 4.2.6 Blood biomarkers

The core of the famous “A/T/N” research framework is to diagnose and predict the AD continuum by using biomarkers to achieve the purpose of early diagnosis (Jack et al., 2018). Although the ATN framework provides a biological definition, which is beneficial to the early diagnosis of the AD continuum and the discovery of pathophysiological mechanisms, there are also some problems. There are dangers associated with the highly invasive operation of cerebrospinal fluid collection. Aβ-PET is costly, only a few institutes have the equipment, and there is a risk of gamma radiation exposure (Shi et al., 2021). These drawbacks prevent it from being widely used for population screening. Therefore, researchers aim to identify a range of novel biomarkers at various levels, especially peripheral biomarkers that are inexpensive, accessible, repeatable, and non-invasive (Simrén et al., 2021).

In this prospect, blood biomarkers have been the focus. In this prospect, blood biomarkers have been the focus. Peripheral marker development has been aided by advancements in novel technologies, including immunomagnetic reduction, next-generation mass spectrometry, single-molecule arrays, and more, which have significantly boosted blood marker detectability (Hirtz et al., 2023). Furthermore, many studies have reported that plasma biomarker models are sensitive to early AD pathology and can accurately identify people at high risk of developing AD (Kivisäkk et al., 2022; Planche et al., 2023). Such evidence suggests that blood-based markers are promising, many plasma markers have been developed in the past decade, such as AD core pathological features (Aβ, tau), glial activation (glial fibrillary acidic protein, Chitinase-3-like protein 1), neurodegeneration (neurofilament light, Micro RNAs), lipid metabolism (heart-type fatty acid binding protein, phosphocholine phosphatase 1), inflammation/chemotaxis (CCL15, CX3CL1, CSF-1, CXCL9, CCL23 and IL-8), etc (Chatterjee et al., 2023).

As previously indicated, these blood biomarkers have great potential as non-invasive means of detecting MCI and provide additional information beyond the ATN framework. What is particularly noteworthy is that sEVs can cross the blood-brain barrier and obtain inaccessible information in brain center, making it one of the most promising biomarkers in MCI detection (Cano et al., 2023). However, several issues limit the development of blood biomarkers. Since this information is not combined with ATN biomarkers, it is insufficient to provide the best explanation for MCI. On the other hand, even though several blood biomarkers have been linked to MCI, not all of them—NF-L, GFAP, and others—may be exclusively associated with MCI, which could lead to incorrect diagnoses. Furthermore, to evaluate biomarkers with precision, existing assays’ detection performance needs to be improved.

Although the topic of MCI has advanced significantly over the past decade owing to the efforts of numerous researchers, there are still a few challenges that need to be solved, necessitating further work in the future. As a supplement to the A/T/N system, new innovative biomarkers associated with the X component are required to increase the accuracy of MCI biological characterization. It is necessary to explore and verify the most effective diagnostic and prognostic model in a sizable prospective cohort.

#### 4.2.7 Integrated management of MCI

There is still a vacuum in medicine for improving cognitive function or possibly reversing the progression of Alzheimer’s disease (Kasper et al., 2020). Therefore, modifiable risk factors—especially lifestyle modifications—are essential. Primary care physicians ought to advise patients and primary caregivers about the available preventative interventions as soon as an MCI diagnosis is made (You, 2024). Lifestyle factors, including cognitive engagement, sleep quality, and nutritional habits, play a crucial role in the potential prevention of MCI. Some studies have found that a balanced dietary management strategy that includes fruits, vegetables, nuts, grains, legumes, olive oil, moderate fish consumption, and a low intake of dairy products, red meat, and meat-based foods can help prevent MCI (He et al., 2024; Lou et al., 2024). Several healthy eating patterns have been shown to offer neuroprotective benefits and should be recommended by primary care physicians, including the Mediterranean diet, the Dietary Approaches to Stop Hypertension (DASH) diet, and the KetoFLEX 12/3 diet (Rao et al., 2023). Besides, a high-quality sleep helps repair brain damage and eliminate waste, whereas sleep disorders limit the removal of beta-amyloid (Aß) and tau, resulting in poorer cognition (Lam et al., 2024). Sleep disturbances have been recognized as a risk factor and an outcome of MCI. Benzodiazepines for insomnia have a higher risk of dementia and should be used with caution. Melatonin can alter the circadian rhythm and boost memory, which can be recommended (Mayer et al., 2024).

## 4.3 Limitations

Only documents from 2013 to 2023 were included in this study. With the no-time limit search strategy, a total of 14,036 records in the WoSCC were searched. However, the enormous of data prevents further visual analysis. Furthermore, there has been significant advancement in the MCI field over the previous ten years. To identify the latest research hotspots and frontiers in MCI, the documents from 2013 to 2023 were chosen as the inclusion criteria.

## 5 Conclusion

The cognition of MCI has been continuously evolved and renewed by multiple countries’ joint efforts in past decade. To promote the development of field of MCI, the close connection between transcontinental countries need to be further strengthened. Hotspots for current research are on diagnostic biomarkers, such as fluid markers, neuroimaging, and so on. Future hotspots might be focused on the best prognostic and diagnostic models generated by machine learning and a large-scale screening tools such as EEG and blood biomarkers.

## Data availability statement

The original contributions presented in this study are included in this article, further inquiries can be directed to the corresponding author.

## Author contributions

CH: Software, Visualization, Writing – original draft. XH: Data curation, Writing – review & editing. MW: Software, Writing – review & editing. XY: Validation, Writing – review & editing. MZ: Supervision, Writing – review & editing. YL: Data curation, Writing – review & editing. LS: Funding acquisition, Supervision, Writing – review & editing. YD: Data curation, Writing – review & editing. ZC: Methodology, Writing – review & editing. HW: Methodology, Writing – review & editing. HS: Methodology, Writing – review & editing.
